# Identification and Functional Characterization of a Novel PRPS1 Variant in X-Linked Nonsyndromic Hearing Loss: Insights From Zebrafish and Cellular Models

**DOI:** 10.1155/humu/6690588

**Published:** 2025-02-14

**Authors:** Yining Wan, Jinqiu Li, Yingyuan Guo, Fang Guo, Ying Zhao, Yue Li, Xia Yang, Huidan Chen, Shimin Xie, Mingyong Wang, Guofang Guan, Yilong Zhu, Xiao Li

**Affiliations:** ^1^Department of Otolaryngology, The Second Hospital of Jilin University, Changchun, Jilin, China; ^2^Academicians Workstation of Jilin Province, Changchun University of Chinese Medicine, Changchun, Jilin, China; ^3^Murui Biological Technology Co. Ltd., Suzhou Industrial Park, Suzhou, Jiangsu, China; ^4^Changchun Veterinary Research Institute, Chinese Academy of Agricultural Sciences, Changchun, Jilin, China

**Keywords:** exome sequencing, novel variant, oxidative stress, PRPS1, X-linked nonsyndromic hearing loss

## Abstract

**Purpose:** The study was aimed at identifying the pathogenic gene responsible for X-linked nonsyndromic hearing loss (NSHL) in a five-generation Chinese family and at elucidating the gene's function both in vivo using a zebrafish model and in vitro using PRPS1 knockdown HEI-OC1 cells.

**Methods:** Exome sequencing (ES) and Sanger sequencing were used to identify the pathogenic variants. A transgenic zebrafish model overexpressing the novel PRPS1 variant (c.494G>A: p.Cys165Tyr) was constructed, and PRPS1 was knocked down in HEI-OC1 cells using siRNA to explore the underlying mechanisms. Hair cell development and behavior were assessed in zebrafish, and mitochondrial function and cell viability were analyzed in HEI-OC1 cells.

**Results:** A novel missense variant (c.494G>A: p.Cys165Tyr) in the PRPS1 gene was identified as the pathogenic variant causing progressive X-linked deafness-1 (DFNX1). The variant led to hair cell death in zebrafish, with disrupted swimming behavior. In HEI-OC1 cells, PRPS1 knockdown resulted in downregulation of the nicotinamide adenine dinucleotide (NAD^+^)/sirtuin 3 (SIRT3)/superoxide dismutase 2 (SOD2) pathway, increased reactive oxygen species (ROS) accumulation, mitochondrial dysfunction, and apoptosis, which were partially rescued by pretreatment with nicotinamide mononucleotide (NMN), a precursor of NAD^+^.

**Conclusion:** The study reports a novel PRPS1 variant contributing to the variant spectrum of PRPS1 and highlights the role of PRPS1 deficiency in increasing oxidative stress-induced hair cell apoptosis via the NAD^+^/SIRT3/SOD2 pathway. These findings provide new insights into the molecular mechanisms of PRPS1-related hearing loss and potential therapeutic targets.

## 1. Introduction

Hearing loss (HL) is a significant global public health issue, with the World Health Organization (WHO) reporting that over 430 million people currently require interventions such as hearing aids to manage disabling HL. This number is expected to rise to over 700 million by 2050. The etiology of HL is diverse, with approximately 60% of cases attributed to genetic factors. Hereditary hearing loss (HHL) is classified into syndromic hearing loss (SHL) and nonsyndromic hearing loss (NSHL), depending on the presence of associated anomalies in other organs. Among NSHL cases, the most common inheritance patterns are autosomal recessive (75%–80%) and autosomal dominant (20%), while X-linked (< 2%) and mitochondrial (< 1%) inheritance patterns are relatively rare [1].

Despite the rarity of X-linked NSHL, five genes have been implicated in its pathogenesis: POU3F4, PRPS1, SMPX, AIFM1, and COL4A6 [2]. The discovery of new pathogenic genes or variants associated with deafness remains a critical area of research in HHL.

In humans, the PRPS1 gene, located on chromosome Xq22.3, encodes phosphoribosyl pyrophosphate synthetase I (PRS-I). This enzyme catalyzes the conversion of ribose-5-phosphate (R5P) to phosphoribosyl pyrophosphate (PRPP), a key substrate for the de novo and salvage pathways of purine, pyrimidine nucleotides, nicotinamide adenine dinucleotide (NAD^+^), and nicotinamide adenine dinucleotide phosphate (NADP^+^) synthesis [3]. Variants in PRPS1 can alter PRS-I activity and PRPP synthesis, leading to disruptions in essential cellular processes such as nucleic acid and energy metabolism. Clinically, the severity of the phenotypes associated with PRPS1 variants generally correlates with the degree of PRS-I activity reduction. Variants that reduce PRS-I activity are linked to several disorders, including X-linked deafness-1 (DFNX1, MIM 304500), X-linked Charcot–Marie–Tooth neuropathy Type 5 (CMTX5, MIM 311070), and Arts syndrome (MIM 301835) [4]. Conversely, variants that increase PRS-I activity have been associated with gout and/or uric acid lithiasis, which may present in isolation or alongside HL and neurological symptoms [5]. Notably, HL is the common feature among these conditions, typically manifesting as postlingual progressive HL. To date, 37 missense variants of PRPS1 have been reported, yet the mechanisms underlying HL associated with these variants remain poorly understood.

Zebrafish (*Danio rerio*) have emerged as a powerful model organism for studying auditory function and HHL due to their genetic and physiological similarities to humans, particularly in inner ear development [6]. The transparency of zebrafish embryos, their rapid development, and the ease of genetic manipulation make them an ideal model for in vivo studies of gene function and associated phenotypes [7]. In the context of hearing research, zebrafish offer unique advantages, including the ability to visualize hair cell function in real time and to conduct high-throughput genetic screens. Previous studies using zebrafish have successfully modeled various forms of HHL, providing valuable insights into the molecular mechanisms underlying these conditions.

Sirtuins (SIRTs), a family of highly conserved NAD^+^-dependent deacetylases, include seven members in mammals, with SIRT3 playing a crucial role in regulating mitochondrial function through deacetylation of various substrates such as superoxide dismutase 2 (SOD2) [8, 9]. Given the role of PRPS1 in NAD^+^ biosynthesis, this study is aimed at further exploring the role of PRPS1 variants in HL, particularly through the NAD^+^/SIRT3/SOD2 pathway, using the cellular model.

Our results demonstrate that prps1a gene knockout (KO) in zebrafish leads to hair cell apoptosis and marked abnormalities in auditory behavior, which can be partially rescued by the introduction of wild-type (WT) human prps1 mRNA. However, the Cys165Tyr variant of prps1 fails to restore normal function, highlighting the critical role of this variant in HL. These findings underscore the utility of zebrafish as a model for studying the genetic basis of auditory function and HL.

## 2. Materials and Methods

### 2.1. Participants and Clinical Assessment

**Figure 1:**
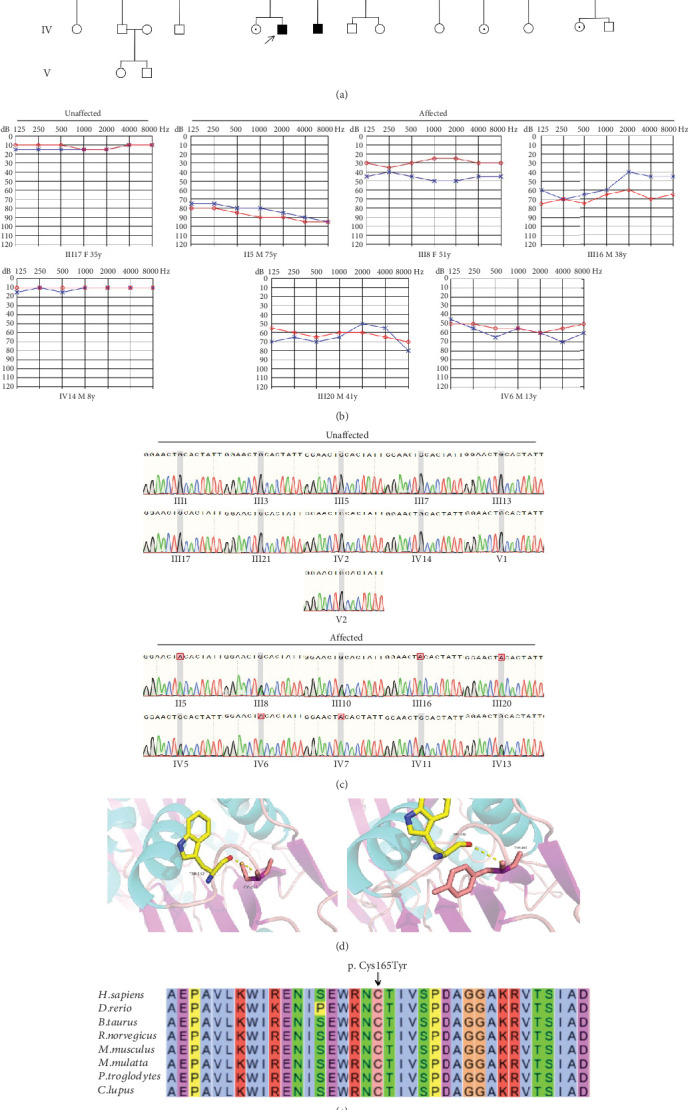
Identification of a novel PRPS1 variant in human hearing loss. (a) Pedigree of a five-generation Chinese family with X-linked HL. Affected males are represented by filled squares, and affected females are represented by circles with center dots. Unaffected individuals are represented by open squares (males) and circles (females). The proband is indicated with a black arrow. Slant lines mark deceased individuals. (b) Audiograms of selected family members, showing air conduction thresholds. Blue “X” denotes the left ear, and red “O” denotes the right ear. (c) Sanger sequencing data from 21 participants showing the normal, hemizygous, and heterozygous PRPS1 variant carriers. (d) Crystal structure of human PRS-I (PDB: 2H06) and the predicted structure with the p.Cys165Tyr variant. (e) Conservation analysis of the cysteine residue at position 165 across species, estimated by T-Coffee.

A five-generation Chinese family with X-linked HHL was identified for this study, comprising a total of 51 individuals. Five individuals with HL and 16 normal-hearing individuals participated in the research. Written informed consent was obtained from all participants, and the study was approved by the Institutional Ethics Committee of the Second Hospital of Jilin University. Clinical data were collected from all participants using a questionnaire that covered gender, age, age of onset, specific clinical manifestations, noise exposure, medication history, and the presence of other systemic diseases such as gout, peripheral neuropathy, optic neuropathy, hypotonia, delayed motor development, ataxia, mental retardation, and recurrent infections. Physical examinations, otoscopy, pure tone audiometry (PTA), and acoustic immittance measurements were conducted to assess the degree of HL and middle ear function. Laboratory tests included blood and urine uric acid levels. The proband (IV-6, Figure 1(a)) underwent additional tests, including auditory brainstem response (ABR), distortion product otoacoustic emission (DPOAE), high-resolution computed tomography (HRCT), and internal auditory canal magnetic resonance imaging (IAC MRI) to determine the etiology of the HL. HL was classified according to the WHO 2021 guidelines as *normal* (< 20 dB), *mild* (20–35 dB), *moderate* (35–50 dB), *moderately severe* (50–65 dB), *severe* (65–80 dB), *profound* (80–95 dB), or *complete* (≥ 95 dB).

### 2.2. Exome Sequencing (ES) and Variant Analysis

Genomic DNA (gDNA) was extracted from the peripheral blood of all participants using standard protocols. The proband (IV-6) underwent ES. gDNA that met the sequencing quality standards (concentration ≥ 12.5 ng/*μ*L, A260/A280 = 1.8–2.0) was randomly fragmented using the M220 Focused-ultrasonicator (Covaris, Woburn, MA, United States). The fragmented gDNA was subjected to 2 × 150 bp paired-end massively parallel sequencing on the MGISEQ-2000 Sequencing System (MGI, Shenzhen, China) following library preparation, hybridization, capture, and purification. Sequencing data were filtered to remove junctions and low-quality bases and then mapped to the human genome reference sequence (GRCh37/hg19) for variant identification. The conservation of the p.C165Y variant was assessed by aligning PRPS1 sequences across species using T-Coffee. The protein structure and function of newly identified variants were predicted and analyzed using AlphaFold2, PyMOL, PolyPhen-2, MutationTaster, and FATHMM to evaluate potential deafness-causing gene variants.

### 2.3. Sanger Sequencing

Sanger sequencing was employed to confirm the segregation of the PRPS1 variant (c.494G>A, p.Cys165Tyr) with the HL phenotype in this family. Sequencing was performed using the ABI 3730xl DNA Analyzer (Life Technologies, Carlsbad, CA, United States), and results were aligned with the transcript NM_002764 in hg19.

### 2.4. PRS-I Activity Measurement

The PRS-I activity measurement was optimized based on previously described methods, which involve detecting adenosine monophosphate (AMP) levels in samples. Hemoglobin was extracted from the peripheral blood of five hemizygotes, five heterozygotes, two unaffected participants, and two unrelated controls. The hemoglobin was diluted fivefold and added to 100 *μ*L of freshly prepared reaction buffer containing 50 mmol/L tris hydrochloride (Tris-HCl) (Sangon Biotech, A610103), 5 mmol/L MgCl_2_ (Sangon Biotech, B601193), 1 mmol/L ethylenediaminetetraacetic acid (EDTA) (Sangon Biotech, A600075), 1 mmol/L Dithiothreitol (DTT) (Sangon Biotech, A620058), 32 mmol/L Na_2_HPO_4_ (Sangon Biotech, A610404), 0.5 mmol/L Adenosine triphosphate (ATP) (Sangon Biotech, A600311), 0.5 mmol/L R5P (Sigma-Aldrich, 83875), and 0.25 mmol/L P1,P5-Di(adenosine-5') pentaphosphate (Ap5A) (Sigma-Aldrich, D6392). The reaction was conducted at 37°C for 30 min. Then, 200 *μ*L of 10% Trichloroacetic acid (TCA) (Sigma-Aldrich, T8657) was added to the reaction buffer. Samples were centrifuged at 17,000 g for 5 min, and 200 *μ*L of supernatant was collected for analysis using a Rigol-L3000 high-performance liquid chromatography (HPLC) system (Rigol, Beijing, China). The system utilized a Sepax HP C18 HPLC column (250 nm × 4.6 mm, 5 *μ*m) with a mobile phase A (MPA) consisting of 10.689 g NaH_2_PO_4_ (Sangon Biotech, A600878) and 11.283 g Na_2_HPO_4_ dissolved in 1 L of water, and a mobile phase B (MPB) consisting of methanol. PRS-I activity was expressed as the generated AMP content (nmol/mL·h).

### 2.5. Zebrafish Maintenance and Husbandry

WT zebrafish (*Danio rerio*) were used in this study, and all experiments were conducted under standard conditions. The zebrafish were maintained in a recirculating water system at 28.5°C with a 14-h light/10-h dark cycle.

### 2.6. Design of gRNA, Activity Verification, and Microinjection

To knock out the prps1a gene in zebrafish, two gRNA target sites were designed. The gRNA sequences were selected using online tools to ensure specificity and efficacy and were synthesized via in vitro transcription. The gRNAs were then complexed with Cas9 protein to form Cas9-gRNA ribonucleoproteins (RNPs). These complexes were microinjected into zebrafish embryos at the 1-cell stage, approximately 1 h postfertilization. Injected embryos were incubated at 28.5°C.

gDNA was extracted from embryos 48 h postinjection to verify the variants induced by the gRNAs. The target region of the prps1a gene was amplified by PCR, and variants were identified and characterized using T7E1 endonuclease digestion and sequencing. WT and Cys165Tyr variant prps1 mRNA were synthesized in vitro, purified, and injected into embryos at the 1-cell stage to assess their effects on hair cell development and auditory behavior.

### 2.7. Hair Cell Staining and Imaging

Hair cell development in zebrafish larvae was assessed using FM1-43FX dye (Thermo Fisher Scientific). The larvae were immersed in an E3 medium containing FM1-43FX for 1 min, followed by three washes in a fresh E3 medium to remove excess dye. The larvae were then embedded in 1.5% low-melting agarose and mounted on slides for imaging. Images were acquired using a Leica TCS SP8 confocal microscope, capturing Z-stack images to cover the entire hair cell layer. Imaging conditions were consistent across all groups, and hair cell counts were analyzed using ImageJ software.

### 2.8. Behavioral Assay

The acoustic startle response in zebrafish is a simple and high-throughput method for assessing auditory function. In this experiment, the Danio Vision behavioral tracking system (Noldus) was used to collect and analyze zebrafish movement data. Broadband noise and baseline noise were edited using Adobe Audition, and the sound signals were emitted through speakers connected to the computer, with noise levels measured by an AS804 noise meter. The experiment setup included baseline noise at 62 dB for 5 min, followed by broadband noise at 96 dB for 15 s, repeated for three cycles.

Before the experiment, 10-day-old zebrafish were placed individually in 24-well plates with 1 mL of system water per well. The experiment was conducted in complete darkness, and the fish were acclimated for 30 min after equipment calibration. The experiment began with baseline noise (62 dB, 5 min), followed by broadband noise (96 dB, 15 s), repeated for three cycles. The Danio Vision system recorded the zebrafish movement trajectory and activity index to assess their response to acoustic stimuli.

### 2.9. Acridine Orange (AO) Staining and Imaging

Hair cell apoptosis in zebrafish larvae was identified by AO dye (Micklin, China). The larvae were immersed in phosphate-buffered saline (PBS) with 5 *μ*g/mL AO, placed in an incubator at 28°C for 20 min, and then washed with PBS three times. Subsequently, the larvae were anesthetized with 0.03% MS-222. For observing apoptosis in hair cells, larvae were excited at a wavelength of 488 nm under a fluorescence microscope (Nikon SMZ800N, Japan). Imaging conditions were consistent across all groups, and hair cell counts were analyzed using ImageJ software.

### 2.10. Cloning of Human prps1 cDNA into pCDNA-3.1myc/His and Synthesis of Capped mRNA

Total RNA was extracted from human cell lines and reverse transcribed into cDNA using reverse transcriptase (Promega). Specific primers were designed to amplify the open reading frame (ORF) of the prps1 gene. The PCR-amplified product was purified and cloned into the pCDNA-3.1myc/His expression vector. The construct was confirmed by restriction enzyme digestion and sequencing.

After validation, the plasmid was used for in vitro transcription using T7 RNA polymerase to synthesize capped mRNA. A cap analog, G(5⁣′)ppp(5⁣′)G (m7GpppG), was included in the reaction to enhance mRNA stability and translation efficiency. The quality and concentration of the synthesized capped mRNA were confirmed by electrophoresis and spectrophotometry, and the mRNA was subsequently used in zebrafish microinjection and functional assays.

### 2.11. Cell Culture

HEI-OC1 cells were cultured in high-glucose Dulbecco's modified Eagle's medium (DMEM, Gibco, United States) supplemented with 10% fetal bovine serum (FBS, Gibco, United States) and incubated under 10% CO_2_ at 33°C. Cells were seeded in T25 cell culture flasks and digested using 0.25% trypsin/EDTA (Hyclone, United States) when the cell density reached 80%.

### 2.12. Drug Administration

*β*-Nicotinamide mononucleotide (NMN, Beyotime, China) was initially dissolved in culture medium to a concentration of 100 mM and then used in subsequent experiments at a final concentration. To evaluate the effects of NMN, cells were pretreated with 100 *μ*M NMN for 24 h before siRNA transfection.

### 2.13. siRNA Transfection

HEI-OC1 cells were transfected with PRPS1-siRNA or negative-siRNA (RIBOBIO, China) at a concentration of 50 nM using INVI DNA RNA Transfection Reagent (Invigentech, United States) according to the manufacturer's instructions. Cells were seeded in 12-well plates and transfected when they reached 70% confluence. The siRNA and transfection reagent were mixed (4 *μ*L each) by pipetting 10–15 times and incubated at room temperature for 10–15 min. The mixture was then added to the cell culture plate. After 24 h, the culture medium was replaced. After 48 h, cells were collected, and the western blot was performed to evaluate the efficiency of knockdown. The target sequence of PRPS1-siRNA was as follows: TCACGCCGCTGACAAACTT.

### 2.14. Cell Viability Assay

Cell viability was assessed using the cell counting kit-8 (CCK-8) assay. Approximately 3000 cells were seeded in a 96-well plate and cultured in the presence or absence of 100 *μ*M NMN for 24 h before PRPS1-siRNA or negative-siRNA transfection. At 48 h post-transfection, 10 *μ*L of CCK-8 reagent (Beyotime, China) was added to each well, and the cells were incubated for 3 h. Absorbance was measured at 450 nm using a microplate reader (TECAN, Switzerland). Each group was evaluated in triplicate.

### 2.15. Flow Cytometry for Annexin V-FITC/PI

The FITC Annexin V Apoptosis Detection Kit (BD Biosciences, United States) was used to identify apoptotic cells. HEI-OC1 cells were seeded in 12-well plates at a density of 1 × 10^5^ cells/well and treated with NMN (100 *μ*M), PRPS1-siRNA, or negative-siRNA as described above. Cells were treated with trypsin without EDTA, collected, washed three times with PBS, and then resuspended in 1X Annexin V binding buffer at a concentration of 1 × 10^5^ cells/mL. Ten microliters of FITC Annexin V/PI dye was added and incubated at room temperature for 15 min. The percentage of apoptotic cells was determined using flow cytometry (BD C6 PLUS, United States) and analyzed with FlowJo 10.8 software. The experiment was repeated three times.

### 2.16. Reactive Oxygen Species (ROS) and Mitochondrial Membrane Potential (MMP) Assay

The level of intracellular ROS was detected using DCFH-DA (Beyotime, China). HEI-OC1 cells were treated as described above, stained with 10 *μ*M DCFH-DA, and incubated for 20 min. Cells were then stained with 10 *μ*L Hoechst 33342 (Beyotime, China) for 5 min, followed by three washes with serum-free cell culture medium. Fluorescent images were captured using a confocal fluorescence microscope (Carl Zeiss AG, Germany). Fluorescent signal intensity was quantified using flow cytometry (BD C6 PLUS, United States) and analyzed with FlowJo 10.8 software.

Tetramethylrhodamine (TMRM, Invitrogen, United States) staining was used to measure MMP. HEI-OC1 cells were stained with 1 ml TMRM staining solution and incubated for 30 min. Cells were then stained with 10 *μ*L Hoechst 33342 (Beyotime, China) for 5 min, followed by three washes with serum-free cell culture medium. Confocal fluorescence microscopy (Carl Zeiss AG, Germany) and flow cytometry (BD C6 PLUS, United States) were used for analysis. All experiments were repeated three times.

### 2.17. Detection of Superoxide Dismutase (SOD) Activity and Quantification of Intracellular NAD^+^

SOD activity was determined using a total SOD assay kit (Beyotime, China) according to the manufacturer's instructions. Intracellular NAD^+^ levels and the NAD^+^/NADH ratio were quantified using an NAD^+^/NADH assay kit (Beyotime, China) following the manufacturer's instructions. All experiments were repeated three times.

### 2.18. Western Blot

HEI-OC1 cells were seeded in six-well plates. After treatment, the culture medium was discarded, and the cells were lysed on ice with radioimmunoprecipitation assay (RIPA) buffer (Beyotime, China) containing protease inhibitor (Beyotime, China). The lysates were centrifuged at 12,000 rpm for 2 min at 4°C. The BCA Protein Assay Kit (Beyotime, China) was used to measure total protein content in the supernatants. Each sample containing 20 *μ*g of total protein was loaded onto sodium dodecyl sulfate polyacrylamide gel electrophoresis (SDS-PAGE) gels and transferred to polyvinylidene fluoride (PVDF) membranes (Cytiva, United States). Membranes were blocked with Quick Blocking Buffer (Beyotime, China) for 40 min at room temperature and then incubated overnight at 4°C with primary antibodies: anti-PRPS1 (Abcam, ab137577, United Kingdom), anti-SIRT3 (Cell Signaling Technology, 5490, United States), anti-SOD2 (Cell Signaling Technology, 13141, United States), anti-Bcl-2 (Proteintech, 1B3F7, China), anti-Bax (Proteintech, 4G5EB, China), and anti-*α*/*β*-Tubulin (Cell Signaling Technology, 2148, United States). Membranes were washed three times with TBST for 10 min each, then incubated with the appropriate secondary antibodies at room temperature for 40 min. Protein bands were visualized using the ECL chromogenic kit (Thermo, United States) and detected with a gel imaging system. The experiment was repeated three times.

### 2.19. Statistical Analysis

All data are derived from three independent experiments and are expressed as mean ± SD. Data analysis was performed using GraphPad Prism 8.3. One-way analysis of variance (ANOVA) was used to compare data among groups. A *p* value of less than 0.05 was considered statistically significant.

## 3. Results

### 3.1. Clinical Characteristics and Variant Analysis

The proband (IV-6, Figure 1(a)), a 13-year-old male, presented at the otology clinic of the Second Hospital of Jilin University with bilateral HL. Comprehensive assessments, including otoscopy, PTA, ABR, acoustic immittance, DPOAE, HRCT, and IAC MRI, were performed to identify the cause of HL. The results indicated that the patient did not pass the DPOAE in either ear. ABR V wave response was normal, but the V wave response threshold was elevated to 70 dB nHL in the left ear and 60 dB nHL in the right ear. PTA revealed severe bilateral SNHL (Figure 1(b)). No abnormalities were detected in otoscopy, acoustic immittance, HRCT, or MRI. A family history of HL was revealed during further inquiries (Figure 1(b)), suggesting a hereditary cause. Consequently, ES was performed on the proband.

ES identified a rare hemizygous variant in the X-linked PRPS1 gene, absent in the hg19 reference genome. Sanger sequencing (Figure 1(c)), laboratory tests, PTA, acoustic immittance, and otoscopy were subsequently performed on additional family members, with 21 individuals participating in total. The results revealed five affected individuals (four males and one female) with HL. Affected males (II-5, III-16, III-20, and IV-6) exhibited bilateral symmetric, postlingual, progressive, severe to profound HL with a flattened audiogram, developing during the first two decades of life. The affected female (III-8) presented with asymmetric moderate HL at age 49, more severe on the left side. Sanger sequencing confirmed the presence of the PRPS1 variant (c.494G>A: p.Cys165Tyr) in all affected individuals. Among the unaffected individuals, four were heterozygotes (III-10, IV-5, IV-11, and IV-13) and one was a hemizygote (IV-7). None had other systemic diseases such as gout, peripheral neuropathy, optic neuropathy, hypotonia, delayed motor development, ataxia, mental retardation, and recurrent infections. PRS-I activity was measured in five hemizygotes, five heterozygotes, two unaffected individuals, and two unrelated controls to assess the functional impact of p.C165Y. The results demonstrated a significant correlation between enzyme activity and HL severity. Compared to healthy participants, five patients with HL exhibited PRS-I activity ranging from 45.68–85.29 nmol/mL·h, while unaffected heterozygotes and hemizygotes showed higher PRS-I activity (277.87–331.22 nmol/mL·h), which was markedly higher than those with HL (*p* < 0.05, ANOVA), but still lower than the healthy persons (*p* < 0.05, ANOVA). This suggests that reduced PRS-I activity contributes to the HL phenotype observed in the family. The characteristics of HL in this family are consistent with the X-linked inheritance pattern associated with DFNX1. The PRPS1 variant (c.494G>A: p.Cys165Tyr) was fully cosegregated with the HL phenotype in this family. Audiological characteristics and PRS-I activity levels for a subset of participants are summarized in Table 1.

PRPS1 encodes PRS-I, and variants in PRPS1 may alter the crystal structure and activity of PRS-I. We used AlphaFold2 and PyMOL to predict the functional impact of the p.C165Y variant on PRS-I enzyme activity. The results indicated that the variant did not affect the active site of PRS-I but altered the local secondary structure (Figure 1(d)). T-Coffee analysis showed a high degree of conservation of cysteine at position 165 across several species (Figure 1(e)). All in silico tools predicted that p.C165Y is likely to be damaging.

### 3.2. Gene KO of prps1a and Its Effects on Zebrafish Hair Cells and Behavior

**Figure 2:**
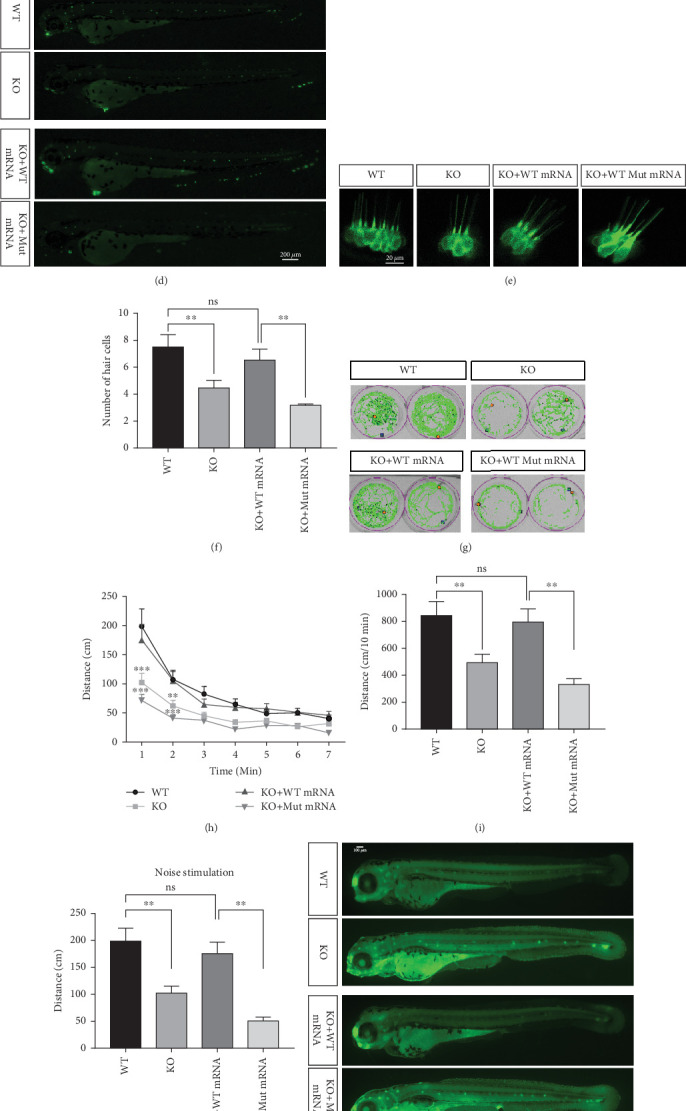
Characterization of prps1a gene editing and phenotypic analysis in zebrafish. (a) Schematic of the zebrafish prps1a gene structure, highlighting the gRNA target sites. Arrows indicate gRNA binding sites, with exons represented as boxes. (b) Sequencing analysis of mutations induced by the two gRNAs showing various mutation types, including insertions, deletions, and substitutions. (c) Validation of gRNA activity through cleavage assays, assessing the efficiency of double-strand break induction by each gRNA. (d) Hair cell staining in zebrafish larvae across four experimental groups: wild-type (WT), gene KO, KO with wild-type mRNA injection (KO+WT mRNA), and WT with Cys165Tyr mutant mRNA injection (WT+Cys165Tyr). *Scale* *bar* = 200*  μ*m. (e) Confocal microscopy images of hair cells in zebrafish larvae from different experimental groups showing structural integrity and localization. *Scale* *bar* = 20*  μ*m. (f) Quantification of hair cells in different experimental groups. (g) Behavioral tracking of zebrafish larvae, recording movement patterns to assess the impact of prps1a gene editing on behavior. (h) Line graph of swimming distance of zebrafish with different genotypes under noise. (i) Statistical analysis of behavioral data of total distance. (j) Statistical analysis of behavioral data of first distance. (k) Representative microscopic images of hair cells stained with AO in zebrafish larvae from different experimental groups. *Scale* *bar* = 100*  μ*m. (l) Quantification of fluorescence intensity in hair cells of the larvae. Data were compared using ANOVA followed by post hoc tests, with significant differences indicated (*p* < 0.05). Data are presented as mean ± SD. ⁣^∗^*p* < 0.05, ⁣^∗∗^*p* < 0.01, ⁣^∗∗∗^*p* < 0.001, and ⁣^∗∗∗∗^*p* < 0.0001.

Zebrafish has two paralogs (prps1a and prps1b). A previous study has shown that zebrafish prps1a mutants and prps1a/prps1b double mutants showed similar morphological phenotypes with reduced hair cell numbers, whereas prps1b mutants showed no overt phenotype [10]. Therefore, we focused our study on the prps1a. In the early stages of this experiment, we designed two gRNAs targeting the zebrafish prps1a gene and validated their activity (Figure 2(a)). Sequencing analysis revealed that after gRNA injection, various variants were induced at the target sites, including insertions, deletions, and substitutions (Figure 2(b)). Further activity assessments confirmed that both gRNAs effectively induced double-strand breaks in the prps1a gene (Figure 2(c)). These results provided a solid foundation for subsequent gene KO experiments.

In the prps1a gene KO group, we observed a significant reduction in the number of hair cells in zebrafish larvae (Figures 2(d), 2(e), and 2(f)), along with disrupted swimming patterns (Figure 2(g)) and a marked decrease in swimming distance (Figures 2(h) and 2(i)). Because the swimming distance after the first noise stimulation is representative, we had extra statistics on this data. The results showed that the distance was also significantly reduced (Figure 2(j)). These findings indicate that the loss of the prps1a gene severely impacts hair cell development and behavior in zebrafish.

When WT prps1 mRNA was injected into the KO group, the number of hair cells partially recovered (Figures 2(d), 2(e), and 2(f)), and zebrafish swimming behavior improved (Figures 2(h), 2(i), and 2(j)). These results suggest that WT prps1 mRNA can effectively rescue hair cell development and behavioral impairments caused by gene KO.

However, when prps1 mRNA carrying the Cys165Tyr mutation was injected, neither the number of hair cells nor behavioral performance recovered, and defects similar to those in the KO group were observed (Figures 2(d), 2(e), 2(f), 2(g), 2(h), 2(i), and 2(j)). This indicates that the Cys165Tyr mutation cannot rescue the phenotypic abnormalities caused by the loss of the prps1a gene. These experiments reveal the crucial role of the prps1a gene in hearing, with Cys165Tyr being a critical site that significantly affects auditory function.

To further investigate the mechanism of prps1a in reducing hair cells, we observed the apoptosis signals of hair cells in the lateral line and inner ear of zebrafish by AO staining. The results showed that hair cell apoptosis signals were significantly increased in the prps1a KO group, which were reduced by the WT prps1 mRNA injection, but no reduction after the Mut prps1 mRNA injection (Figures 2(k) and 2(l)). We preliminarily speculated that the PRPS1 gene may cause HL by inducing hair cell apoptosis.

### 3.3. PRPS1 Knockdown Reduces NAD^+^ Levels, NAD^+^/NADH Ratio, and Cell Viability in HEI-OC1 Cells

**Figure 3:**
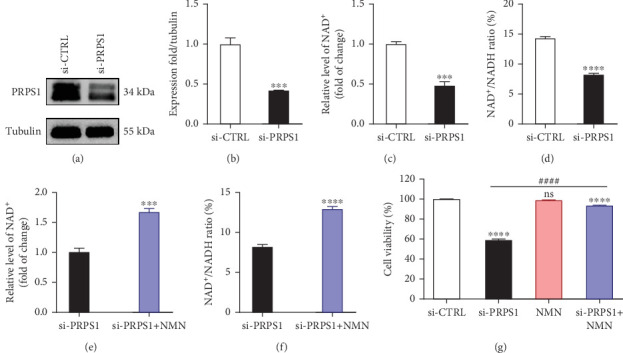
PRPS1 knockdown reduces NAD^+^ levels, NAD^+^/NADH ratio, and cell viability in HEI-OC1 cells. (a) Western blot analysis of PRPS1 expression after transfection with PRPS1-siRNA or negative-siRNA, with tubulin as the internal control. (b) Quantification of PRPS1 expression levels from (a). Measurement of intracellular (c) NAD^+^ levels and (d) NAD^+^/NADH ratio in si-CTRL and si-PRPS1 groups. Measurement of intracellular (e) NAD^+^ levels and (f) NAD^+^/NADH ratio in si-PRPS1 and si-PRPS1+NMN groups. (g) Cell viability assessed by CCK-8 assay. Data are presented as three independent experiments' mean ± SD. ⁣^∗∗∗^*p* < 0.001 and ⁣^∗∗∗∗^*p* < 0.0001 compared with the si-CTRL; ^####^*p* < 0.0001 compared with the si-PRPS1.

The HEI-OC1 cell line, derived from the auditory organ of the mouse, is a progenitor hair cell line widely used as an in vitro research model [11]. To explore the underlying mechanisms of PRPS1 in HL, we knocked down PRPS1 in HEI-OC1 cells using PRPS1-siRNA (si-PRPS1) and compared the results to cells transfected with negative-siRNA (si-CTRL). Western blot analysis confirmed the efficiency of PRPS1 knockdown, showing significantly reduced PRPS1 expression after PRPS1-siRNA transfection (Figures 3(a) and 3(b)). PRPP, catalytically generated by PRS-I, serves as an intermediate in NAD^+^ biosynthesis. Previous studies have shown that reduced PRS-I activity decreases NAD^+^ production, impairing cell proliferation, which can be partially rescued by NAD^+^ replenishment [12]. We hypothesized that PRPS1 deficiency might affect hair cell viability by downregulating NAD^+^. We measured intracellular NAD^+^ content and the NAD^+^/NADH ratio after transfection. The results indicated a significant decrease in both NAD^+^ levels (Figure 3(c)) and the NAD^+^/NADH ratio (Figure 3(d)) in si-PRPS1 cells compared to si-CTRL cells. Pretreatment with *β*-NMN, a precursor of NAD^+^, effectively increased intracellular NAD^+^ content and the NAD^+^/NADH ratio (Figures 3(e) and 3(f)). Cell viability assays using CCK-8 showed a significant decrease in cell activity following PRPS1 knockdown, which was partially rescued by NMN supplementation (Figure 3(g)). These findings suggest that NAD^+^ is essential for mitigating the deafness phenotype associated with PRPS1 deficiency. Thus, subsequent experiments were conducted with four groups: HEI-OC1 cells transfected with negative-siRNA (si-CTRL), PRPS1-siRNA (si-PRPS1), si-CTRL pretreated with NMN (NMN), and si-PRPS1 pretreated with NMN (si-PRPS1+NMN).

### 3.4. PRPS1 Knockdown Regulates Intracellular ROS Levels and Mitochondrial Function via the NAD^+^/SIRT3/SOD2 Pathway in HEI-OC1 Cells

**Figure 4:**
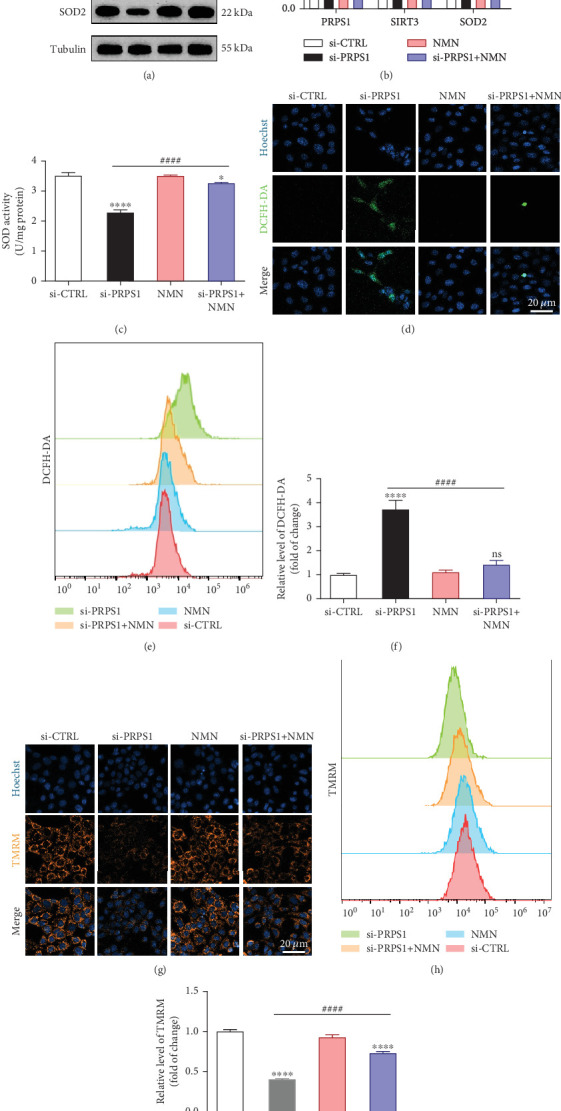
PRPS1 knockdown regulates intracellular ROS levels and mitochondrial function via the NAD^+^/SIRT3/SOD2 pathway in HEI-OC1 cells. (a) Western blot analysis of PRPS1, SIRT3, and SOD2, with tubulin as the internal control. (b) Quantification of protein expression levels from (a). (c) SOD activity measured using a total superoxide dismutase assay kit with WST-8. (d) Representative microscopic images of HEI-OC1 cells stained with DCFH-DA in each group. *Scale* *bar* = 20 *μ*m. (e, f) Flow cytometry analysis of DCFH-DA staining, showing quantitative changes in green fluorescence. (g) Representative microscopic images of HEI-OC1 cells stained with TMRM in each group. *Scale* *bar* = 20*  μ*m. (h, i) Flow cytometry analysis of TMRM staining, showing quantitative changes in fluorescence. Data are presented as mean ± SD from three independent experiments. ⁣^∗^*p* < 0.05, ⁣^∗∗∗^*p* < 0.001, and ⁣^∗∗∗∗^*p* < 0.0001 compared with si-CTRL; ^####^*p* < 0.0001 compared with si-PRPS1.

NAD^+^ is a coenzyme in multiple redox reactions, and as an NAD^+^-dependent deacetylase, mitochondrial SIRT3 enhances mitochondrial antioxidative capacity by deacetylating enzymes such as SOD2 [13], thereby reducing ROS production and protecting cells from oxidative stress. We investigated whether PRPS1 knockdown influences intracellular ROS levels and mitochondrial function via the NAD^+^/SIRT3/SOD2 pathway in HEI-OC1 cells. Western blotting showed that PRPS1 knockdown reduced SIRT3 and SOD2 protein levels (Figures 4(a) and 4(b)), while NMN treatment significantly increased their expression. SOD activity assays revealed that PRPS1 knockdown reduced SOD activity, which could be partially restored by NMN pretreatment (Figure 4(c)). Intracellular ROS levels were measured using DCFH-DA staining, and both immunohistochemistry (Figure 4(d)) and flow cytometry (Figures 4(e) and 4(f)) indicated a significant increase in ROS production following PRPS1 knockdown. NMN treatment significantly reduced ROS levels in the si-PRPS1+NMN group compared to si-PRPS1 alone. Mitochondrial dysfunction was assessed by measuring MMP using TMRM staining. Immunohistochemistry (Figure 4(g)) and flow cytometry (Figures 4(h) and 4(i)) showed a marked reduction in MMP levels in the si-PRPS1 group, which was partially rescued by NMN pretreatment.

### 3.5. PRPS1 Knockdown Increases HEI-OC1 Cell Apoptosis

**Figure 5:**
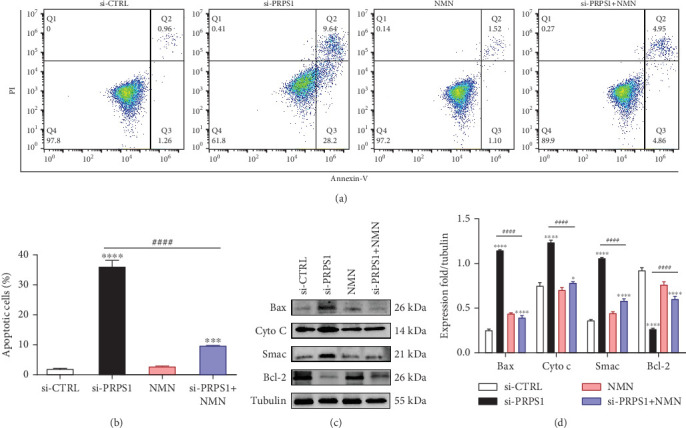
PRPS1 knockdown increases apoptosis in HEI-OC1 cells. (a, b) Apoptotic cells detected by flow cytometry in each group. (c) Western blot analysis of proapoptotic proteins Bax, Cytochrome c, Smac/Diablo, and antiapoptotic protein Bcl-2, with tubulin as the internal control. (d) Quantification of protein expression levels from (c). Data are presented as mean ± SD from three independent experiments. ⁣^∗∗∗^*p* < 0.001 and ⁣^∗∗∗∗^*p* < 0.0001 compared with si-CTRL; ^####^*p* < 0.0001 compared with si-PRPS1.

Numerous studies have shown that ROS accumulation and mitochondrial dysfunction lead to hair cell apoptosis [14, 15]. To assess the effect of PRPS1 knockdown on hair cell apoptosis, Annexin V-FITC/PI assays were conducted to measure the proportion of apoptotic cells. The results showed a significant increase in apoptosis in the si-PRPS1 group (37.84%), which was reduced to 9.81% by NMN pretreatment (Figures 5(a) and 5(b)). Western blot analysis confirmed the upregulation of proapoptotic proteins Bax, Cytochrome c, and Smac/Diablo, and the downregulation of the antiapoptotic protein Bcl-2 in the si-PRPS1 group, with these changes being significantly reversed by NMN pretreatment (Figures 5(c) and 5(d)). Collectively, these findings suggest that mitochondrial dysfunction via the NAD^+^/SIRT3/SOD2 pathway is a key mechanism underlying PRPS1-associated HL.

## 4. Discussion

The field of HHL research is advancing rapidly. To date, more than 200 loci associated with HHL have been mapped, and at least 124 genes have been cloned, providing a foundation for diagnosing and preventing HHL. However, it is estimated that approximately 50% of the genes responsible for deafness remain undiscovered. Identifying new pathogenic genes or novel variants of known genes is crucial for advancing our understanding of HHL. In this study, we examined a five-generation Chinese family with X-linked NSHL and identified a novel variant (c.494G>A: p.Cys165Tyr) in the PRPS1 gene through ES, which resulted in reduced PRS-I activity and consequently DFNX1. This variant has not been previously reported, and the mechanisms by which PRPS1 variants cause deafness remain unclear. To investigate this, we developed a prps1a KO zebrafish model and conducted knockdown experiments in HEI-OC1 cells. Our findings provide in vivo and in vitro evidence that PRPS1 deficiency leads to hair cell damage, potentially through downregulation of mitochondrial function via the NAD^+^/SIRT3/SOD2 axis.

The PRPS1 gene, located on Xq22.3, encodes PRS-I. Missense variants in PRPS1 can result in increased or decreased PRS-I activity, leading to disorders such as PRS-I superactivity, DFNX1, CMTX5, and Arts syndrome [16]. The first DFNX1 family was reported in 1996, presenting with congenital profound NSHL [17]. However, all subsequently reported DFN2 families presented with postlingual progressive NSHL, consistent with X-linked inheritance, where affected males presented with early-onset, severe to profound HL, while female heterozygotes had either symmetric or asymmetric HL, ranging from mild to moderate after age 40 [18]. In our study, the affected individuals had HL without other systemic diseases such as gout, peripheral neuropathy, optic neuropathy, hypotonia, delayed motor development, ataxia, mental retardation, and recurrent infections. Therefore, we concluded that the variant (c.494G>A: p.Cys165Tyr) leads to a DFNX1 phenotype, with most male hemizygotes exhibiting severe to profound HL, except for a 4-year-old child who did not develop HL. Among the female heterozygotes, only one developed HL at the age of 49, while others maintained normal hearing. Enzyme activity assays revealed that individuals without HL had higher PRS-I activity compared to those with HL, though still lower than in healthy controls. The higher PRS-I activity in heterozygotes may result from compensation by the second X chromosome. However, the variation in enzyme activity, particularly in a 49-year-old female heterozygote and a 4-year-old male hemizygote, remains unexplained. Tissue-specific regulatory mechanisms, such as the role of edited microRNA-376 in PRPS1 expression [19], could contribute to the progressive decrease in PRS-I activity and subsequent HL, warranting further investigation. Our study identified a novel missense variant (c.494G>A: p.Cys165Tyr) in PRPS1, which led to DFNX1, and we suggested that PRS-I activity may serve as a blood biomarker for PRPS1-related diseases. Furthermore, long-term monitoring of enzyme activity in affected individuals could guide clinical counseling.

Zebrafish have emerged as a vital model organism in studying human diseases due to their transparent embryos, rapid development, and ease of genetic manipulation. In hearing research, zebrafish are particularly valuable because their inner ear structure closely resembles that of mammals, making them an ideal model for exploring the mechanisms of hereditary deafness [20]. Zebrafish has two paralogs (prps1a and prps1b). Both paralogs display remarkable similarity to human PRPS1, with an identity of approximately 97%. Furthermore, DeSmidt et al. revealed that prps1 is expressed in the otic vesicle of zebrafish larvae using whole mount in situ hybridization [6]. Pei et al. demonstrated that zebrafish prps1a and prps1b are relatively enriched in the inner ear, and they found that the decrease in the number of neuromast hair cells was primarily due to the loss of prps1a expression [10]. This evidence indicated that prps1a plays a vital role in maintaining the zebrafish hair cell's function. In light of these studies, we designed the gRNA target site to knock out the prps1a gene in zebrafish, and WT and Cys165Tyr variant prps1a mRNA were injected into embryos at the 1-cell stage to assess their effects on hair cell development and auditory behavior. Our results showed that prps1a gene KO in zebrafish led to a significant reduction in hair cells and abnormalities in behavioral indicators. Injection of WT prps1 mRNA partially restored these defects, whereas mRNA carrying the Cys165Tyr mutation failed to do so. These findings underscore the importance of the Cys165Tyr site in maintaining PRPS1 gene function.

The PRPS1 gene is highly conserved across species, highlighting its essential role in fundamental cellular functions. Research has shown that PRPS1 is critical not only in zebrafish but also in other species, being associated with various hereditary diseases, including NSHL and neurological disorders [10]. This evolutionary conservation enhances our understanding of the gene's function across different species. Our findings in zebrafish are consistent with human genetic studies, where the Cys165Tyr mutation is strongly linked to progressive HL. The validation of the PRPS1 gene and its critical site using the zebrafish model provides new insights into the mechanisms of HHL and emphasizes the gene's importance in auditory function.

Previous studies have highlighted that maintaining the abundance of NAD^+^ levels in hair cells plays a prominent role in alleviating age-related hearing loss (ARHL), noise-induced hearing loss (NIHL), and ototoxic HL. These occur by correcting mitochondrial dysfunction, oxidative stress, DNA damage, and inflammatory response [21, 22]. At present, the most widely used NAD^+^ precursors are nicotinic acid (NA), nicotinamide (NAM), nicotinamide riboside (NR), and NMN [23]. NA and NAM are primarily employed in the treatment of pellagra. However, it should be noted that these medications can cause side effects such as flushing and GI disturbances [24, 25]. NR is transformed to NMN by Nicotinamide riboside kinases (NRKs), and NRK2 is mainly present in cardiac and skeletal muscle tissues, indicating that NR treatment increases the NAD^+^ level in a tissue-specific manner [26]. In this study, we used NMN to investigate whether a reduction in NAD^+^ levels is a potential mechanism underlying PRPS1-related deafness. We found that PRPS1 knockdown in HEI-OC1 cells decreased intracellular NAD^+^ levels and cell viability, effects that were reversed by NMN pretreatment. These findings indicate that NMN supplementation can mitigate the adverse effects of PRPS1 knockdown, highlighting the potential of NAD^+^ precursors in treating PRPS1-associated HL.

SIRT3, a NAD^+^-dependent deacetylase, is known to regulate mitochondrial function and ROS levels by deacetylating antioxidant enzymes such as SOD2. Mitochondrial dysfunction and ROS accumulation are linked to increased degeneration of spiral ganglion neurites and hair cell apoptosis [27, 28]. Our results are consistent with these findings, as PRPS1 knockdown reduced SIRT3 and SOD2 expression, increased ROS production, and disrupted MMP. NMN pretreatment alleviated these effects, enhancing MMP and reducing ROS levels. Furthermore, apoptosis assays confirmed that the prps1a gene KO would increase the apoptosis of zebrafish inner ear and lateral hair cells in vivo. And in vitro, PRPS1 knockdown increased HEI-OC1 cell apoptosis, which was attenuated by NMN. These results confirmed that PRPS1 deficiency caused the reduction of NAD^+^ synthesis. The decrease in NAD^+^ levels led to the decrease of SIRT3 (NAD^+^-dependent deacetylase) expression and activity, as well as the decrease of its substrate SOD2 expression and activity. This resulted in a reduction in the antioxidant capacity of the mitochondria, an accumulation of ROS, and an increase in apoptosis. Thus, our study explores the role of the deafness gene PRPS1 in HL, particularly through the NAD^+^/SIRT3/SOD2 pathway.

## 5. Conclusions

In summary, we identified a novel missense variant (c.494G>A: p.Cys165Tyr) in PRPS1 associated with X-linked HL in a five-generation Chinese family. Our transgenic zebrafish model overexpressing this variant provided further validation of its pathogenicity. Our findings suggest that PRPS1 knockdown causes mitochondrial dysfunction, increases ROS accumulation, and induces hair cell apoptosis, potentially through the NAD^+^/SIRT3/SOD2 pathway. Future research will focus on developing a PRPS1 variant (c.494G>A: p.Cys165Tyr) model in zebrafish and HEI-OC1 cells to comprehensively characterize the variant's role in deafness.

## Figures and Tables

**Table 1:** Summary of the clinical data of a part of participants from the family.

**Subject**	**Sex**	**Test (year)**	**Onset (year)**	**Sanger sequencing**	**HL severity**	**PRS-I activity (nmol/mL·h)**
II-5	M	75	15	Hemizygote	Profound	45.68 (±5.49)
III-8	F	51	49	Heterozygote	Moderate	85.29 (±7.16)
III-10	F	35	—	Heterozygote	—	297.88 (±13.33)
III-16	M	38	11	Hemizygote	Severe	52.58 (±3.42)
III-17	F	35	—	Normal	—	351.20 (±21.44)
III-20	M	41	10	Hemizygote	Severe	57.97 (±4.53)
IV-5	F	25	—	Heterozygote	—	331.22 (±32.69)
IV-6	M	13	13	Hemizygote	Severe	83.90 (±6.72)
IV-7	M	4	—	Hemizygote	—	277.87 (±26.17)
IV-11	F	11	—	Heterozygote	—	294.62 (±19.72)
IV-13	F	13	—	Heterozygote	—	329.01 (±29.78)
IV-14	M	8	—	Normal	—	348.26 (±31.56)
Ctrl1	F	45	—	Normal	—	355.31 (±20.88)
Ctrl2	M	26	—	Normal	—	361.23 (±18.33)

## Data Availability

The data that support the findings of this study are available from the corresponding authors upon reasonable request.
